# Therapeutic benefits of intravenous cardiosphere-derived cell therapy in rats with pulmonary hypertension

**DOI:** 10.1371/journal.pone.0183557

**Published:** 2017-08-24

**Authors:** Ryan C. Middleton, Mario Fournier, Xuan Xu, Eduardo Marbán, Michael I. Lewis

**Affiliations:** 1 Heart Institute, Cedars-Sinai Medical Center, Los Angeles, CA, United States of America; 2 Division of Pulmonary/Critical Care Medicine, Cedars-Sinai Medical Center, Los Angeles, CA, United States of America; University of Kansas Medical Center, UNITED STATES

## Abstract

Pulmonary arterial hypertension (PAH) is a progressive condition characterized by occlusive pulmonary arteriopathy, in which survival remains poor despite pharmacologic advances. The aim of this study was to evaluate the ability of cardiosphere-derived cells (CDCs), cardiac progenitor cells with potent anti-inflammatory and immunomodulatory properties, to attenuate hemodynamic and morphometric remodeling of the right ventricle (RV) and pulmonary arterioles in rats with established monocrotaline (MCT)-induced PAH. Animals were divided into 3 groups: 1) Control (CTL), 2) PAH in which CDCs were centrally infused (CDC) and 3) PAH in which saline was given (Sham). Significant increments in RV systolic pressure (RVSP) and RV hypertrophy were noted in Sham animals compared to CTL. In CDC rats at day 35, RSVP fell (- 38%; p< 0.001) and RV hypertrophy decreased (-26%; p< 0.01). TAPSE and cardiac output were preserved in all 3 groups at day 35. Pulmonary arteriolar wall thickness was greater in Sham rats compared to CTL, and reduced in CDC animals for vessels 20–50 μm (P<0.01; back to CTL levels) and 50–80μm (P<0.01) in diameter. The macrophage population was increased in Sham animals compared to CTL (P< 0.001), but markedly reduced in CDC rats. In conclusion, infusion of CDCs markedly attenuated several key pathophysiologic features of PAH. As adjunctive therapy to PAH-specific agents, CDCs have the potential to impact on the pathobiology of adverse pulmonary arteriolar remodeling, by acting on multiple mechanisms simultaneously.

## Introduction

The pathobiology of pulmonary arteriolar hypertension (PAH) involves endothelial cell injury and dysfunction, smooth muscle cell proliferation, matrix alterations, and degradation culminating in occlusive arteriolar remodeling [1]. Furthermore, inflammation and immune dysfunction have emerged as key contributors to the pathogenesis of vascular remodeling [2] Occlusive arteriopathy results in increased right ventricular (RV) afterload that commonly progresses to RV dysfunction and failure [3]. Even with substantial pharmacologic advances, survival in PAH remains unacceptably poor. In the large French registry, the 3-year survival in incident cases was only 54.9% [4]. Recent data from the US REVEAL registry [5] reported 5-year outcomes for functional class I, II, III and IV in prevalent cases to be 88%, 75.6%, 57% and 27.2% respectively. Of note, only 27% of patients actually improve their functional class [6] Further, despite clinical improvement on PAH-specific medications, progressive RV dysfunction [3], persistent severe occlusive arteriopathy, and plexiform lesions still occur [7]. Thus, an adjunctive therapy that could directly address and attenuate severe occlusive arteriopathy is greatly needed. This rationale has been the basis for studies of gene- and cell-based therapies in animal models of PAH [8].

Cardiosphere-derived cells (CDCs) are heart-derived progenitor cells that exhibit multilineage potential and clonogenicity. As with other stem cell lineages, such as bone marrow derived mesenchymal stem cells (BM-MSCs), CDCs also express endoglin (CD105), a TGF-β receptor subunit. However, unlike BM-MSCs, CDC do not express the pan-hematopoietic marker, CD45. CDCs [9]. CDC treatments in models of ischemic heart disease show the regenerative abilities of CDCs by promoting cardiomyocyte proliferation, along with potent anti-inflammatory and immunomodulatory properties and other salutary attributes, including attenuation of fibrosis, apoptosis and oxidative/ nitrosative stress. CDCs also attract local stem cells to sites of injury [10–13]. CDC therapeutic effects are conferred primarily through the secretion of extracellular vesicles, including exosomes [14].

In this study, we sought to evaluate the impact of CDCs administered by central intravenous infusion on hemodynamic and structural responses of the RV, and pulmonary arteriolar remodeling, in animals with established PAH. In preliminary studies, we demonstrated a reduction in right ventricular systolic pressure (RVSP) and RV hypertrophy in PAH rats in which CDCs were administered by central intravenous infusion (S3 Fig). Based on this finding, we tested the hypothesis that reduced RVSP might result from favorable vascular remodeling, possibly related to suppression of cellular inflammation by CDCs.

## Methods

### Animals

Adult male Sprague-Dawley (Charles River) rats were fed Purina rat chow and water ad libitum. Animals were individually housed with a dark: light cycle of 12 hours under ambient temperature. For surgical procedures and echocardiography measurements, rats were anaesthetized using 2–5% isoflurane. The protocol was approved by the Cedars-Sinai Animal Care and Use Committee.

### Induction of PAH: Monocrotaline model

**Fig 1:**
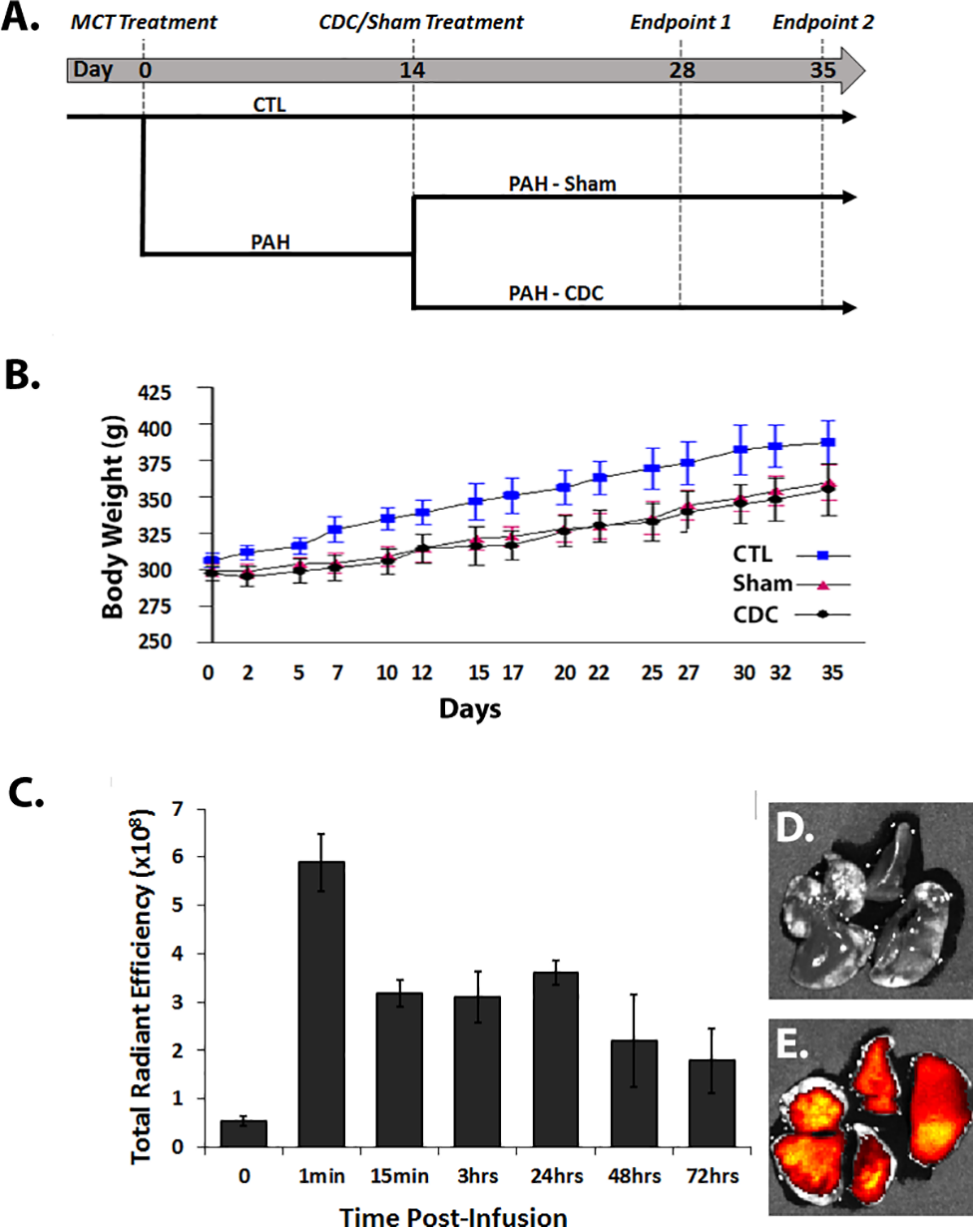
Experimental design and animal characterization. A) Experimental design; 28 days: CTL, n = 10; SHAM, n = 11; CDC, n = 10. 35 days: CTL, n = 6; SHAM, n = 9; CDC, n = 10. B) Body weight (g) measurements taken over the course of the study. C) Time course of the total radiant efficiency of fluorescently-labelled CDC in excised lung. Measurements are from the left lung; 3 animals per group. Representative images of the left and right lungs untreated (D; t = 0) or having received CDC treatment (E; t = 15min).

As shown in Fig 1A, rats were randomly divided into two initial groups: 1) healthy control animals (CTL; n = 16) and 2) Pulmonary Arterial Hypertension (PAH; n = 40) animals. PAH was induced by a single subcutaneous injection of monocrotaline (MCT;60 mg/kg in 0.5mL of phosphate-buffered saline (PBS)), while CTL received only PBS. The PAH rats were then further divided at random into two groups: a) CDC-treated animals (CDC; n = 20) and b) those that received PBS infusion only (Sham; n = 20)

### CDC generation and delivery

Rat CDCs were generated from wild-type strain-matched rat hearts [15]. Cardiac explants (1mm^3^) isolated from the ventricles and septum were cultured for one week, and the fibroblast-like cells that emerged from the explants were trypsinized and plated on low-attachment culture dishes for 72 hours to allow the cardiospheres to form. Then, the cardiospheres in suspension were collected and plated onto fibronectin-coated dishes. The proliferating cardiosphere-derived cells (CDCs) were harvested when ~80% confluency was reached and the CDCs were expanded until passage five (P5). A description of rat CDC markers have been previously described [9]. Fourteen days after MCT-administration, animals received 2 million CDCs (dose based on retention studies; see S2 Fig) in 1mL of PBS (PAH group) or 1 mL of PBS only (Sham), intravenously through a cannula in the external jugular vein over a period of 2 minutes. Terminal experiments were performed at either 28 or 35 days post-MCT administration.

### CDC retention quantification

An additional 21 rats were dedicated to short-term experiments to measure pulmonary CDC retention to provide further insights to our initial retention studies (see Online Supp) in which a different methodology was employed. CDCs were stained with a far-red fluorescent, lipophilic dye (DiD, Thermo Scientific) for 30 min at 37°C and washed to remove free-floating dye. Following CDC infusion, the rats were euthanized at increasing time points (from 1 minute to 72 hours), and the lungs collected in cold PBS. The lungs were imaged using the Xenogen IVIS Imaging system (Ex/Em: 647/665nm; Perkin Elmer) and total radiant efficiency was recorded.

### Echocardiography

Transthoracic two-dimensional, M-mode echocardiography and pulsed-wave Doppler imaging were performed on anesthetized rats (Vevo 770 Micro-Ultrasound imaging system; Visual Sonics: Toronto, Canada). RV systolic function was determined by tricuspid annular plane systolic excursion (TAPSE), recorded in M-mode. Doppler of pulmonary outflow measured pulmonary artery flow velocity time (PA VTI). To obtain stroke volume (SV), the cross-sectional area of the pulmonary artery was multiplied by PA VTI, and estimated cardiac output (CO) was derived by multiplying SV by heart rate. Studies were performed on days 14 (prior to CDC infusion), 28 and 35. Researchers were blinded to treatment group during the acquisition and analysis of echocardiographic data.

### Hemodynamic studies

Under general anesthesia (isoflurane), the right external jugular vein was exposed surgically and cannulated with a 1.4F Mikro-Tip catheter pressure transducer (SPR-671; Millar Instruments, Houston, TX). The catheter was advanced into the RV and tip pressure was measured via a dual channel pressure control unit (PCU-2000; Millar Instruments).

### Right ventricular morphometrics

After hemodynamic studies, the heart was excised, the RV wall was separated from the LV and the interventricular septum (S) using a dissecting microscope, and weights recorded. Both RV mass and RV/(LV+S) weights (the Fulton Index) were used as indices of RV hypertrophy. Researchers were blinded to treatment group during the acquisition and analysis of hemodynamics and right ventricular morphometrics.

### Lung immunohistology and cell identification

#### Arteriolar wall thickness

Both lungs were rapidly excised and preserved with 4% paraformaldehyde. Segments from multiple lobes were mounted in paraffin and 5μm transverse sections were cut and mounted onto glass slides for immunohistology. To evaluate arteriolar smooth muscle thickness, slides were incubated in rabbit anti-smooth muscle α-actin (1:200, Abcam) in blocking reagent (Dako), and subsequently incubated in donkey anti-rabbit Alexa Fluor 546 (1:400, Life Technologies) and mounted onto coverslips using FluoroShield™ with DAPI mounting medium (Sigma). Sections were imaged using the TCS SP5 II confocal microscope (Leica Biosystems, Vista, CA). Vessels with diameters 20–110μm were analyzed for wall thickness index, i.e. the total outer vessel area minus the luminal area, divided by total outer vessel area (5 animals per group; 60–250 vessels per animal counted from 10–15 images, taken randomly throughout lung tissue).

#### Peri-vascular lung macrophage infiltration

To evaluate macrophage infiltration, lung tissue sections were stained with a mouse anti-rat CD68 primary antibody (1:200, Sigma), followed by goat anti-mouse Alexa Fluor 488 secondary antibody. Smooth muscle actin (SMA) and DAPI were also applied and imaged as described above (5 animals per group, 13–20 images per animal, taken randomly throughout lung tissue). Only macrophages within a 250μm radius from a SMA+ vessel were counted. For both arteriolar wall thickness and macrophage infiltration studies, researchers were blinded for the imaging and analysis of the lung histology experiments.

### Statistical analysis

Data were tested for normality and statistical analysis performed using ANOVA to compare differences between independent groups. If a significant interaction was found, post hoc analysis (Student-Newman-Keuls test) was used to compare differences between independent groups. An α level of 0.05 was used to compare differences between groups and overall significance. Values are expressed as means ± SE.

## Results

### Animal groups

Male rats were randomly divided into two initial groups with 40 animals receiving a single dose of MCT (PAH) to induce pulmonary hypertension, and 16 animals receiving PBS (CTL). The PAH rats were then further divided at random into two groups two weeks later; one group received 2 million CDCs by intravenous infusion into the jugular vein and the other group received PBS by the same method (Fig 1A). For details regarding the initial studies for CDC dose determination and timing of CDC infusion, see S2 Fig.

### Body weights

Fig 1B shows serial body weights for the 3 groups which were measured on alternate days. The Sham and CDC groups were comparable. Of note, the body weights of Sham and CDC rats both diverged from CTL over the first two weeks after monocrotaline, after which all groups gained weight in parallel.

### CDC lung retention

To determine the retention rate of CDCs within the lungs following intravenous delivery, fluorescently-labelled CDCs were infused and total radiant efficiency was measured from lungs excised at increasing timepoints. Lung retention of CDCs was highest immediately following intravenous infusion and remained stable from 15 minutes to 24 hours, after which CDC detection decreased (pooled data in Fig 1C and representative fluorescence images in Fig 1D and 1E).

### RV hemodynamics and morphometry

RV systolic pressure (RVSP) is significantly elevated in patients with PAH as well as animals that have received monocrotaline. A progressive increase in RVSP leads to adaptive RV remodeling(hypertrophy) of the right ventricle. To assess the effects of CDCs in PAH animals, RVSP and RV morphometry were measured 28 and 35 days post-MCT infusion.

#### 28-day data

**Fig 2:**
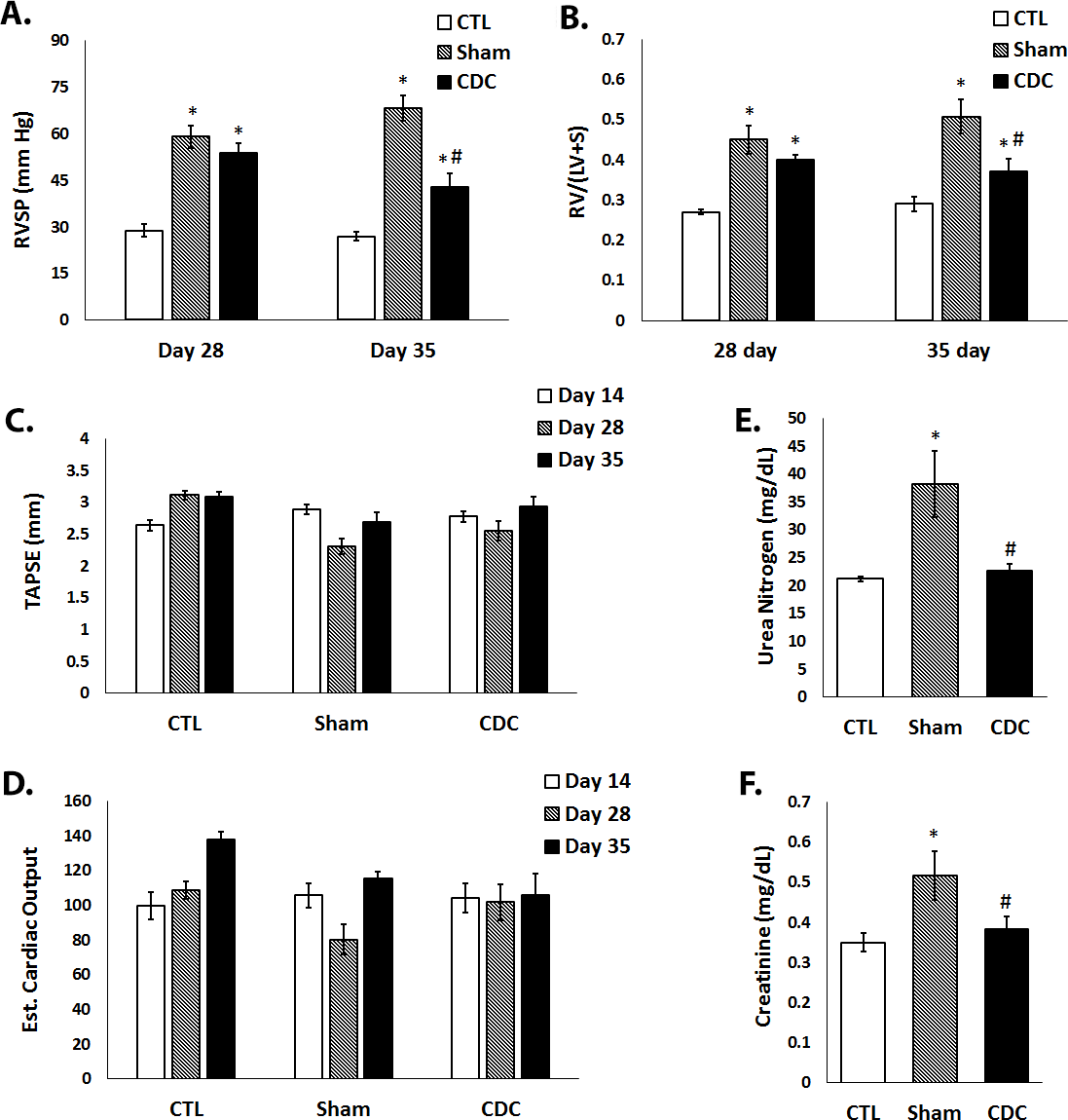
Hemodynamic and morphometric studies. (A) Right ventricular systolic pressure (RVSP) and (B) Fulton Index in control animals (CTL) and animals with PAH (Sham and CDC) 28 days and 35 days following MCT administration. (C) Tricuspid Annular Plane Systolic Excursion (TAPSE) and (D) estimated cardiac output (CO) at day 28 and day 35. Values depicted as means ± SEM. E) Blood Urea Nitrogen (BUN) and F) serum creatinine levels (mg/dL). * significantly different from CTL. # significantly different from Sham treatments.

MCT induced a 210% increase in RVSP at 28 days (p< 0.001 vs. CTL; Fig 2A). By contrast, PAH animals that received CDCs exhibited a 9.5% decrease in RVSP as compared to Sham PAH rats. At 28 days (Fig 2B), the Fulton index (a measure of RV hypertrophy) in Sham rats increased 178% (p<0.01 vs. CTL), but this value was reduced by 11.1% in CDC-treated PAH rats as compared to Sham PAH rats.

#### 35-day data

At day 35, RVSP increased further in Sham (239% increment vs. CTL; p <0.001; Fig 2A). By 35 days, CDC treatment prevented further increase in RVSP compared to Sham rats (now attenuated by 38%; p<0.001 vs. Sham). A similar increment in the Fulton index was noted at 35 days (Fig 2B) in Sham (180% increment; p<0.01). By contrast, the index fell by 26% in CDC rats at day 35 (p<0.01), approaching levels similar to that seen in CTL.

### Echocardiography

Neither TAPSE (a commonly used measure of RV systolic function) nor echocardiography-derived cardiac output (CO) showed any systematic differences among groups at either 28 or 35 days (Fig 2C and 2D). Thus, in MCT-induced PAH, at both time frames and with treatment of CDCs or vehicle, RV pump function was preserved. Progressive decrements in these two indices were noted in MCT-treated animals after 42 days post MCT administration indicative of right heart failure (preliminary data, not shown). Thus, we can confidently state that in the present study, RV pump function was preserved at both 28 and 35 days post MCT administration and that pump failure could not account for the decline in RSVP observed with CDC administration.

#### Arteriolar wall thickening

**Fig 3:**
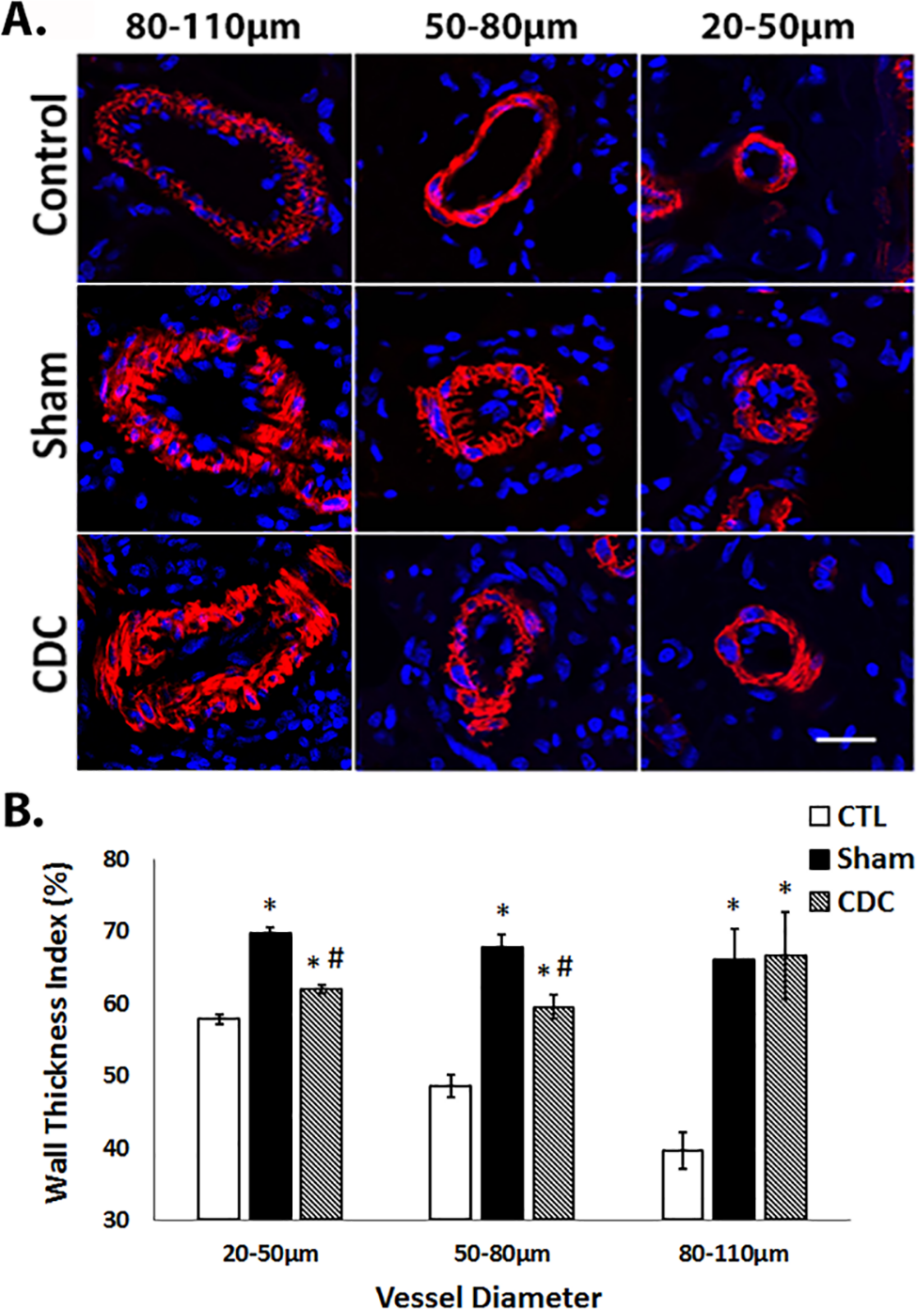
Mean vessel wall thickness for the 3 treatment groups. (A) Immunohistochemical depictions of pulmonary arterioles for each of the 3 treatment groups, with size based on outer vessel diameter. Lung tissue sections were stained with alpha smooth muscle actin (red) and DAPI (blue). (B) Graphical representation of the vessel wall thickness index for each treatment group (n = 5 per group). Scale bar = 25μm. Values depicted as means ± SEM. * significantly different from CTL; # significantly different from Sham treatments.

To investigate the potential mechanism for the reduction in RVSP and RV hypertrophy in CDC-treated animals, we analyzed pulmonary arteriolar vessel wall thickness in all three groups. Based on robust and extensive literature on inflammation as a key early factor in PAH pathobiology, we proposed that the known potent anti-inflammatory properties of CDCs would act upon key mechanisms of arteriolar remodeling, to reduce arteriolar thickening and thus RV remodeling. Sham animals showed increased wall thickness in small (20–50μm), medium (50–80 μm) and large (80–110 μm) vessels (p<0.001 vs. CTL; Fig 3A and 3B). Central infusion of CDCs led to decreased pulmonary arteriolar wall thickness in the small and medium vessel groups, compared to Sham (p<0.001), but there were no detectable differences between Sham and CDC in large vessels.

#### Macrophage infiltration

**Fig 4:**
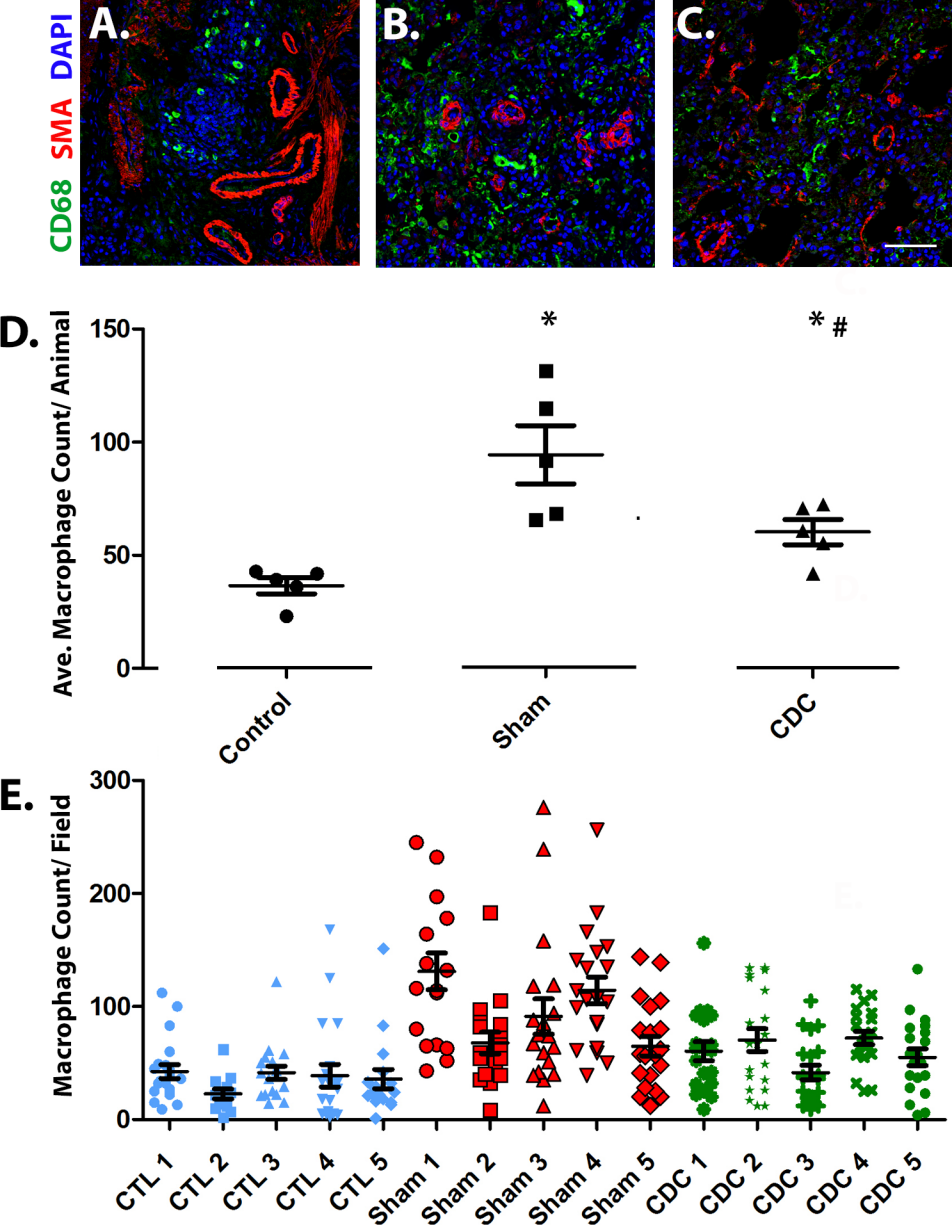
Macrophage infiltration assessment for the 3 treatment groups. Representative images of lung histology from (A) CTL (B) Sham and (C) CDC- treated animals. Images display the macrophage marker CD68 (green), smooth muscle actin (red) and DAPI (blue). Scale bar = 50μm. (D) Graphical representation of the average macrophage count within the lung for each treatment group (n = 5 per group). Values depicted as means ± SEM. * significantly different from CTL; # significantly different from Sham treatments. (E) Graphical representation of the average macrophage count per field for each animal. (5 per group; 15–20 images per animal).

Macrophages can potentiate smooth muscle cell proliferation associated with PAH through the secretion of cytokines, chemokines and leukotrienes [16,17] Based on reported potent anti-inflammatory effects of CDCs, we assessed macrophage infiltration in the lungs, via immunohistochemistry, at 35 days post CDC or Sham treatments (Fig 4). Sham animals had increased macrophages throughout the peri-vascular areas of the lung and within vessel walls (p< 0.003 vs. control), but macrophage infiltration was attenuated in CDC-treated animals (p<0.02 vs. Sham, Fig 4).

#### Safety and survival data

For both the 28 and 35-day cohorts, there were only 5 premature deaths (8%); far fewer than reported in previous publications [18]. Of the deaths, 3 were in sham PAH animals and 2 in those receiving CDCs. No evidence of hypoxemia or any observable adverse effects were noted at any dose in the 24 hours following CDC infusion. The rats exhibited completely normal activity and behaviors for both investigators and veterinary staff. Arterial Blood Gas measurements taken at 24 hours post CDC infusion, as well as biochemical and hematologic data taken at the 35 day time point, revealed no adverse effects of CDC treatment (S1–S4 Tables). Interestingly, CDC-treated rats exhibited improved renal function as reflected by reduced blood urea nitrogen (BUN) and creatinine levels compared to Sham, reaching the normal levels seen in the CTL group (Fig 2E and 2F).

## Discussion

The administration of CDCs to animals with established PAH was effective in reducing RVSP and RV hypertrophy, in association with improved pulmonary arteriolar morphometry. No adverse effects were evident; in fact, CDCs markedly reduced macrophage infiltration in lung tissue and improved biomarkers of renal function in rats with PAH. The fact that CO and TAPSE (a functional measure of RV systolic function) were preserved and similar to control animals, suggests that the hemodynamic and morphometric improvements, following CDC treatment, are best explained by a fall in pulmonary vascular resistance / afterload facing the RV due to a reduction in occlusive arteriopathy. This has important clinical implications, as occlusive arteriopathy and plexiform lesions are still prominent in patients treated with PAH-specific agents [7], and progressive RV dysfunction can occur despite apparent symptomatic responses to modern PAH-specific agents [3]. Inflammation and immune dysfunction are key early drivers of PAH pathobiology [2]. The monocrotaline model of PAH used in this study is one in which inflammation-driven pulmonary vascular remodeling is prominent. As in patients with PAH, an intense perivascular pulmonary artery inflammatory cell infiltrate is present which includes macrophages, dendritic cells and lymphocytes [17,19]. Inflammatory changes precede the development of pulmonary vascular remodeling [20]. This suggests that inflammation and immune dysfunction are a cause and not the result of vascular disease [2,20].

In the MCT model, increased expression of IL-6 and IL-1β including adventitial IL-6 expressing cells are reported [19], as well as chemokines such as fractalkine [21] and MCP-1. Of interest, LeHiress [22] reported that MCT-induced PAH was reversed with the administration of anti-CD74 antibodies, which blocked the pro-inflammatory cytokine MIF. Further, the beneficial effects of induced pluripotent stem cells (iPCs) in MCT-induced PAH appear to be due to anti-inflammatory properties of the iPCs leading to suppression of NF-κB phosphorylation [23]. Infiltrating macrophages have also been shown to express high levels of leukotriene B4 (LTB4) which facilitate apoptosis of EC and proliferation of PASMC. Blocking LTB4 in the established MCT model reversed PH and improved survival [16] and based on this, a Phase 2 clinical trial is in progress (NCT02664558).

Further, in common with the various forms of PAH [20], the MCT model also displays well-described medial hypertrophy, neointimal proliferation and adventitial changes, EC injury [24,25], increased endothelin-1, downregulation of NO signaling, impaired vasoreactivity and pronounced disruption in BMP and TGF-β signaling linked to increased macrophage recruitment, and inflammation-induced IL-6 expression associated with impaired BMPR2 function [17,26,27].

Notable here was the efficacy of cell therapy in a pre-clinical model of PAH without the need for enhancing transduction of genes such as eNOS [28]. Additionally, studies employing non-transduced CDCs by our group were shown to benefit small- and large-animal models of heart disease [10–13,29,30]. The issue of gene transduction of stem cells is an important one when attempting to bring the “bench to bedside”, as gene transduction would incur incur huge regulatory hurdles to be overcome in combining gene therapy and stem cell therapies when applying for INDs from the FDA. Using CDCs alone, without gene transfection, markedly reduces these hurdles and makes manufacture under GMP conditions orders of magnitude less complicated. Indeed we have IND approval and a 4-year Phase 1 trial in progress to treat patients with PAH with centrally infused CDCs (ClinicalTrials.gov Identifier: NCT03145298; ALPHA Study).

While the specific mechanisms of how CDC treatment resulted in reduced vessel wall thickness are not clear, we postulate that its multiple potent properties impacted on key pathophysiological processes [29,31]. For example, CDCs modulate immune cell behavior and attenuate inflammatory signaling, which are key early drivers of PAH pathophysiology [2,11,12]. Our data are in keeping with a marked anti-inflammatory impact of CDCs in the monocrotaline PAH model through the attenuation of macrophage infiltration. Further, CDCs cause distinctive anti-inflammatory polarization of rat bone marrow-derived macrophages [11] and increase regulatory T-cells [32]. Additionally, CDCs attenuate both oxidative and nitrosative stress (a common form of inflammatory injury), and are potently anti-fibrotic (which impacts the severity of established or ongoing pathobiology [12]. CDCs have also been shown to be anti-apoptotic (acting on early endothelial cell injury to prevent apoptosis and emergence of apoptotic-resistant clones) and can attract endogenous stem cells to sites of vascular injury (acting on early EC injury). Of interest, a head-to-head comparison of 4 different cell types (CDCs, bone marrow-derived mononuclear cells, bone marrow-derived mesenchymal stem cells (MSCs) and adipose-derived MSCs) demonstrated superiority of CDCs in paracrine secretion, angiogenesis, cell differentiation and functional variables in the same mouse infarct model [33]. This attests to the potency of the CDCs which was clearly evident in our current study.

The rationales for timing of rescue therapies and evaluation of responses to CDCs are as follows. In earlier studies, we and others [20] established that the MCT model induced significant elevation in RVSP by day 14 post MCT administration, as well as established arteriopathy, as reported in the literature [34]. Further, in recent studies in which MSCs were given as rescue in animal models of PAH, the cells were also administered 2 weeks after MCT injection [35,36]. The rationale for the selected time points in the present study for determining the efficacy of CDCs was based in part on the range of time frames reported for a large number of studies (>20) in which stem cells were given to rats in which MCT was used to induce PAH [28,35–37]. Additionally, no significant influences on RVSP and Fulton index were observed 10 days after the administration of CDCs (i.e. day 24 post MCT administration; S3 Fig), in preliminary studies. By contrast at day 28, significant changes started to emerge. In keeping with our earlier observations, an enhanced positive impact was demonstrated over time. The study was not extended longer than 35 days, which was determined *a priori* as part of the experimental design, as prior studies had shown a high potential for the development of right heart failure, significant weight loss and associated excessive mortality after 35 days [18].

In conclusion, the use of CDCs in the MCT model of PAH significantly reduced RVSP, RV hypertrophy, pulmonary arteriolar wall thickness and macrophage infiltration. In the modern era of PAH-specific therapies, PAH remains an incurable and progressive disease which exhibits persistent occlusive arteriopathy and progression to RV dysfunction and failure on current therapies. An adjunctive therapy that has the potential to impact on the pathobiology of adverse pulmonary arteriolar remodeling, by acting on multiple mechanisms simultaneously, would be a great advance and CDCs have great potential to fill this niche.

## Supporting information

S1 Fig Serial hemodynamics and indices of RV hypertrophy. Right Ventricle Systolic Pressure (RVSP) (A) and Fulton Index (B) of control (CTL) and PAH animals at days 0, 7, 14, 24, and 28. Note significant gradual increments in RV systolic pressures and increases in the Fulton index at days 14, 24, and 28 in PAH animals compared to CTL animals. Values are means ± SEM; * significantly different from CTL; • significantly different from day 7 PAH; • significantly different from day 14 PAH. All experiments were performed in triplicate.
(DOCX)

S2 Fig Initial CDC dosing and retention studies. CDC retention rate (A) and absolute number of CDC cell engraftment in rat lung tissue (B) following 0.5, 1 and 2 million CDCs infusions into the right external jugular vein. Quantitative PCR was used to quantify the abundance of the SRY gene of male rat-derived CDCs within female rat lung tissue, 24 hours post infusion. All experiments were performed in triplicate.
(DOCX)

S3 Fig RV indices in CDC- or Sham-treated PAH rats. RVSP (A) and Fulton Index (B) for each of the three treatment groups at day 24 (10 days post administration of CDCs). Animals received two million CDCs in PBS or a PBS sham treatment via right external jugular vein injection. All experiments were performed in triplicate.
(DOCX)

S1 Table Arterial blood gases (ABG). Arterial blood gases were drawn 24 hours post infusion of cells under general anesthesia on room air (RA).
(DOCX)

S2 Table Hematology assay. Complete blood count (CBC) and blood chemistry from each of the 3 treatment groups at Day 35.
(DOCX)

S3 Table Biochemistry panel assays, Day 28. (DOCX)

S4 Table Biochemistry panel assays, Day 35. (DOCX)
