# Supplementary material for: Therapeutic benefits of intravenous cardiosphere-derived cell therapy in rats with pulmonary hypertension
Source: PLoS One. 2017 Aug 24;12(8):e0183557. doi: 10.1371/journal.pone.0183557 (PMC5570343; doi:10.1371/journal.pone.0183557)
Supplement: S1 Fig — Right Ventricle Systolic Pressure (RVSP) (A) and Fulton Index (B) of control (CTL) and PAH animals at days 0, 7, 14, 24, and 28. Note significant gradual increments in RV systolic pressures and increases in the Fulton index at days 14, 24, and 28 in PAH animals compared to CTL animals. Values are means ± SEM; * significantly different from CTL; • significantly different from day 7 PAH; • significantly different from day 14 PAH. All experiments were performed in triplicate. (DOCX) [file pone.0183557.s001.docx]

**SUPPLEMENTAL FIGURES**

**S1 Fig. Serial Hemodynamics and indices of RV hypertrophy**

**B**

**A**

**S1. Serial hemodynamics and indices of RV hypertrophy**

Right Ventricle Systolic Pressure (RVSP) (A) and Fulton Index (B) of control (CTL) and PAH animals at days 0, 7, 14, 24, and 28. Note significant gradual increments in RV systolic pressures and increases in the Fulton index at days 14, 24, and 28 in PAH animals compared to CTL animals. Values are means ± SEM; * significantly different from CTL; • significantly different from day 7 PAH; • significantly different from day 14 PAH. All experiments were performed in triplicate.
