# Supplementary material for: Therapeutic benefits of intravenous cardiosphere-derived cell therapy in rats with pulmonary hypertension
Source: PLoS One. 2017 Aug 24;12(8):e0183557. doi: 10.1371/journal.pone.0183557 (PMC5570343; doi:10.1371/journal.pone.0183557)
Supplement: S2 Fig — CDC retention rate (A) and absolute number of CDC cell engraftment in rat lung tissue (B) following 0.5, 1 and 2 million CDCs infusions into the right external jugular vein. Quantitative PCR was used to quantify the abundance of the SRY gene of male rat-derived CDCs within female rat lung tissue, 24 hours post infusion. All experiments were performed in triplicate. (DOCX) [file pone.0183557.s002.docx]

**SUPPLEMENTAL FIGURES**

**S2 Fig. Initial CDC dosing and retention studies**

**Cells/mg of lung tissue**

**B**

**Retention Rate**

**(% of Injected cells)**

**A**

**S2. Initial CDC dosing and retention studies**

CDC retention rate (A) and absolute number of CDC cell engraftment in rat lung tissue (B) following 0.5, 1 and 2 million CDCs infusions into the right external jugular vein. Quantitative PCR was used to quantify the abundance of the SRY gene of male rat-derived CDCs within female rat lung tissue, 24 hours post infusion. All experiments were performed in triplicate.
