# Supplementary material for: Therapeutic benefits of intravenous cardiosphere-derived cell therapy in rats with pulmonary hypertension
Source: PLoS One. 2017 Aug 24;12(8):e0183557. doi: 10.1371/journal.pone.0183557 (PMC5570343; doi:10.1371/journal.pone.0183557)
Supplement: S3 Fig — RVSP (A) and Fulton Index (B) for each of the three treatment groups at day 24 (10 days post administration of CDCs). Animals received two million CDCs in PBS or a PBS sham treatment via right external jugular vein injection. All experiments were performed in triplicate. (DOCX) [file pone.0183557.s003.docx]

**SUPPLEMENTAL FIGURES**

**S3 Fig. RV indices in CDC- or Sham-treated PAH rats**


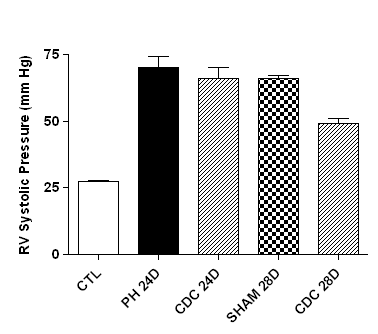


**SHAM**

**A**


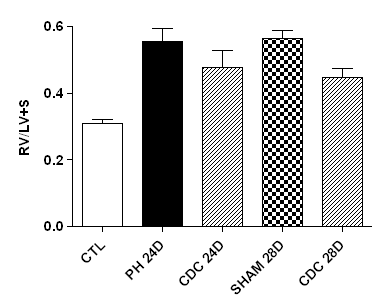


**SHAM**

**B**

*

*

**S3. RV indices in CDC- or Sham-treated PAH rats**

RVSP (A) and Fulton Index (B) for each of the three treatment groups at day 24 (10 days post administration of CDCs). Animals received two million CDCs in PBS or a PBS sham treatment via right external jugular vein injection. All experiments were performed in triplicate.
