# Supplementary material for: Therapeutic benefits of intravenous cardiosphere-derived cell therapy in rats with pulmonary hypertension
Source: PLoS One. 2017 Aug 24;12(8):e0183557. doi: 10.1371/journal.pone.0183557 (PMC5570343; doi:10.1371/journal.pone.0183557)
Supplement: S1 Table — Arterial blood gases were drawn 24 hours post infusion of cells under general anesthesia on room air (RA). (DOCX) [file pone.0183557.s004.docx]

**SUPPLEMENTAL TABLES**

**S1 Table. Arterial Blood Gases (ABG)**

| **CDC Number** | pH | PCO2 | PO2 | HCO3 | | SaCO2 |
| --- | --- | --- | --- | --- | --- | --- |
|  |  | mmHg | mmHg | mM | | % |
| 0.5M | 7.36 | 51.8 | 79 | 29.5 | 95 | |
| 0.5M | 7.42 | 42.8 | 96 | 27.8 | 98 | |
| 1M | 7.33 | 55.7 | 76 | 29.5 | 94 | |
| 2M | 7.41 | 43.6 | 95 | 27.1 | 97 | |
| 2M | 7.46 | 39.7 | 86 | 28.3 | 97 | |
| 2M | 7.39 | 44.8 | 73 | 28 | 94 | |
| 2M | 7.48 | 36.7 | 90 | 27.1 | 98 | |

**S1 Table. Arterial Blood Gases (ABG)**

Arterial blood gases were drawn 24 hours post infusion of cells under general anesthesia on room air (RA).
