# Supplementary material for: Therapeutic benefits of intravenous cardiosphere-derived cell therapy in rats with pulmonary hypertension
Source: PLoS One. 2017 Aug 24;12(8):e0183557. doi: 10.1371/journal.pone.0183557 (PMC5570343; doi:10.1371/journal.pone.0183557)
Supplement: S2 Table — Complete blood count (CBC) and blood chemistry from each of the 3 treatment groups at Day 35. (DOCX) [file pone.0183557.s005.docx]

**SUPPLEMENTAL TABLES**

**S2 Table. Hematology assay**

|  | WBC | Hemoglobin | Hematocrit | Platelet Count |
| --- | --- | --- | --- | --- |
|  | CMM | Mg/dL | % | 1000U/L |
| **CTL** |  |  |  |  |
|  | 5.2 | 12.2 | 39.8 | 595 |
|  | 6.4 | 11 | 35.7 | 646 |
|  | 3.9 | 13 | 41.8 | 641 |
| Mean | 5.17 | 12.07 | 39.1 | 627 |
| SD | 1.25 | 1.01 | 3.11 | 28 |
| **SHAM** |  |  |  |  |
|  | 7 | 13.7 | 42 | 655 |
|  | 9.8 | 14 | 43.5 | 465 |
|  | 9.3 | 14.2 | 44.5 | 534 |
| Mean | 8.7 | 13.97 | 43.33 | 551 |
| SD | 1.49 | 0.25 | 1.26 | 96 |
| **CDC** |  |  |  |  |
|  | 8.6 | 14.4 | 45.1 | 661 |
|  | 7 | 13.9 | 43.7 | 607 |
|  | 5.4 | 13 | 42.5 | 752 |
| Mean | 7 | 13.77 | 43.77 | 673 |
| SD | 1.6 | 0.71 | 1.3 | 73 |

**S2 Table. Hematology Assay**

Complete blood count (CBC) and blood chemistry from each of the 3 treatment groups at Day 35.
