# Supplementary material for: Therapeutic benefits of intravenous cardiosphere-derived cell therapy in rats with pulmonary hypertension
Source: PLoS One. 2017 Aug 24;12(8):e0183557. doi: 10.1371/journal.pone.0183557 (PMC5570343; doi:10.1371/journal.pone.0183557)
Supplement: S3 Table — (DOCX) [file pone.0183557.s006.docx]

**S3 Table. Biochemistry panel assays, Day 28**

|  | **Glucose** | **Sodium** | **Potassium** | **Chloride** | **Carbon Dioxide** | **Anion Gap** | **Urea Nitrogen** | **Creatinine** |
| --- | --- | --- | --- | --- | --- | --- | --- | --- |
|  | mg/dL | mM | mM | mM | mM | MEQ/L | mg/dL | mg/dL |
| **CTL** |  |  |  |  |  |  |  |  |
|  | 278.0 | 140.0 | 4.2 | 100.0 | 25.0 | 15.0 | 19.0 | 0.2 |
|  | 406.0 | 138.0 | 6.2 | 97.0 | 17.0 | 24.0 | 18.0 | 0.4 |
|  | 256.0 | 141.0 | 4.3 | 103.0 | 23.0 | 15.0 | 15.0 | 0.2 |
| Mean | 313.3 | 139.7 | 4.9 | 100.0 | 21.7 | 18.0 | 17.3 | 0.3 |
| SD | 81.0 | 1.5 | 1.1 | 3.0 | 4.2 | 5.2 | 2.1 | 0.1 |
| **SHAM** |  |  |  |  |  |  |  |  |
|  | 233.0 | 140.0 | 4.8 | 100.0 | 28.0 | 12.0 | 19.0 | 0.3 |
|  | 240.0 | 142.0 | 5.1 | 100.0 | 28.0 | 14.0 | 18.0 | 0.3 |
|  | 236.0 | 141.0 | 5.0 | 100.0 | 27.0 | 14.0 | 22.0 | 0.3 |
| Mean | 236.3 | 141.0 | 5.0 | 100.0 | 27.7 | 13.3 | 19.7 | 0.3 |
| SD | 3.5 | 1.0 | 0.2 | 0.0 | 0.6 | 1.2 | 2.1 | 0.0 |
| **CDC** |  |  |  |  |  |  |  |  |
|  | 205.0 | 141.0 | 5.1 | 99.0 | 28.0 | 14.0 | 20.0 | 0.3 |
|  | 208.0 | 140.0 | 5.1 | 99.0 | 26.0 | 15.0 | 22.0 | 0.3 |
|  | 275.0 | 139.0 | 5.4 | 98.0 | 27.0 | 14.0 | 20.0 | 0.3 |
| Mean | 229.3 | 140.0 | 5.2 | 98.7 | 27.0 | 14.3 | 20.7 | 0.3 |
| SD | 39.6 | 1.0 | 0.2 | 0.6 | 1.0 | 0.6 | 1.2 | 0.0 |
|  | **Calcium (Serum)** | **Bilirubin (Total)** | **Total Protein** | **Albumin** | **Alk. Phos.** | **AST** | **ALT** | **Calcium (serum)** |
|  | mg/dL | mg/dL | g/dL | g/dL | U/L | U/L | U/L | mg/dL |
| **CTL** |  |  |  |  |  |  |  |  |
|  | 10.3 | 0.2 | 4.9 | 3.3 | 125.0 | 104.0 | 87.0 | 10.3 |
|  | 11.8 | 0.2 | 5.1 | 3.6 | 175.0 | 197.0 | 128.0 | 11.8 |
|  | 9.7 | 0.2 | 4.9 | 3.3 | 133.0 | 108.0 | 65.0 | 9.7 |
| Mean | 10.6 | 0.2 | 5.0 | 3.4 | 144.3 | 136.3 | 93.3 | 10.6 |
| SD | 1.1 | 0.0 | 0.1 | 0.2 | 26.9 | 52.6 | 32.0 | 1.1 |
| **SHAM** |  |  |  |  |  |  |  |  |
|  | 10.2 | 0.2 | 6.0 | 3.6 | 156.0 | 66.0 | 69.0 | 10.2 |
|  | 10.3 | 0.2 | 5.7 | 3.7 | 128.0 | 179.0 | 166.0 | 10.3 |
|  | 10.8 | 0.2 | 5.7 | 3.5 | 160.0 | 67.0 | 84.0 | 10.8 |
| Mean | 10.4 | 0.2 | 5.8 | 3.6 | 148.0 | 104.0 | 106.3 | 10.4 |
| SD | 0.3 | 0.0 | 0.2 | 0.1 | 17.4 | 65.0 | 52.2 | 0.3 |
| **CDC** |  |  |  |  |  |  |  |  |
|  | 10.5 | 0.2 | 5.8 | 3.7 | 115.0 | 73.0 | 88.0 | 10.5 |
|  | 10.7 | 0.2 | 5.8 | 3.7 | 166.0 | 95.0 | 100.0 | 10.7 |
|  | 10.3 | 0.2 | 5.2 | 3.4 | 133.0 | 109.0 | 99.0 | 10.3 |
| Mean | 10.5 | 0.2 | 5.6 | 3.6 | 138.0 | 92.3 | 95.7 | 10.5 |
| SD | 0.2 | 0.0 | 0.3 | 0.2 | 25.9 | 18.1 | 6.7 | 0.2 |
