# Supplementary material for: Therapeutic benefits of intravenous cardiosphere-derived cell therapy in rats with pulmonary hypertension
Source: PLoS One. 2017 Aug 24;12(8):e0183557. doi: 10.1371/journal.pone.0183557 (PMC5570343; doi:10.1371/journal.pone.0183557)
Supplement: S4 Table — (DOCX) [file pone.0183557.s007.docx]

**S4 Table. Biochemistry panel assays, Day 35**

|  | **Glucose** | **Urea Nitrogen** | **Creatinine** | **Bilirubin (Total)** | **Total Protein** | **Albumin** | **AST** | **ALT** |
| --- | --- | --- | --- | --- | --- | --- | --- | --- |
|  | mg/dL | mg/dL | mg/dL | mg/dL | g/dL | g/dL | U/L | U/L |
| **CTL** |  |  |  |  |  |  |  |  |
|  | 362 | 21 | 0.4 | 0.2 | 7.2 | 4.5 | 82 | 84 |
|  | 367 | 21 | 0.3 | 0.2 | 5.7 | 3.7 | 67 | 71 |
|  | 377 | 20 | 0.3 | 0.2 | 5.4 | 3.6 | 84 | 74 |
|  | 313 | 23 | 0.3 | 0.2 | 5.3 | 3.5 | 62 | 47 |
|  | 393 | 20 | 0.4 | 0.2 | 5.4 | 3.6 | 79 | 68 |
|  | 402 | 22 | 0.4 | 0.2 | 7.2 | 4.5 | 78 | 58 |
| MEAN | 370.4 | 21.17 | 0.35 | 0.2 | 6.03 | 3.9 | 75.33 | 67 |
| SD | 34.85 | 1.17 | 0.05 | 0 | 0.91 | 0.47 | 8.8 | 12.93 |
| **SHAM** |  |  |  |  |  |  |  |  |
|  | 225 | 59 | 0.6 | 0.2 | 4.9 | 2.9 | 135 | 68 |
|  | 288 | 23 | 0.4 | 0.2 | 5.4 | 3.6 | 77 | 45 |
|  | 366 | 48 | 0.7 | 0.2 | 7 | 4.1 | 90 | 77 |
|  | 323 | 45 | 0.6 | 0.2 | 6.6 | 4 | 95 | 70 |
|  | 301 | 26 | 0.3 | 0.2 | 5.4 | 3.6 | 100 | 82 |
|  | 430 | 28 | 0.5 | 0.2 | 6.9 | 4.3 | 76 | 75 |
| MEAN | 322.17 | 38.17 | 0.52 | 0.2 | 6.03 | 3.75 | 95.5 | 69.5 |
| SD | 70.15 | 14.55 | 0.15 | 0 | 0.9 | 0.5 | 21.6 | 13 |
| **CDC** |  |  |  |  |  |  |  |  |
|  | 346 | 23 | 0.4 | 0.2 | 5.6 | 3.8 | 91 | 66 |
|  | 394 | 25 | 0.5 | 0.2 | 6.7 | 4.2 | 105 | 100 |
|  | 285 | 21 | 0.3 | 0.2 | 5.8 | 3.7 | 64 | 60 |
|  | 352 | 23 | 0.4 | 0.2 | 5 | 3.4 | 73 | 66 |
|  | 349 | 26 | 0.4 | 0.2 | 7 | 4.4 | 73 | 72 |
|  | 324 | 18 | 0.3 | 0.2 | 5.6 | 3.6 | 87 | 76 |
| MEAN | 341.67 | 22.67 | 0.38 | 0.2 | 5.95 | 3.85 | 82.17 | 73.33 |
| SD | 35.89 | 2.88 | 0.08 | 0 | 0.75 | 0.38 | 14.97 | 14.18 |
